# Extracts of* Coreopsis tinctoria *Nutt. Flower Exhibit Antidiabetic Effects via the Inhibition of *α*-Glucosidase Activity

**DOI:** 10.1155/2016/2340276

**Published:** 2016-03-21

**Authors:** Wujie Cai, Lijing Yu, Yu Zhang, Li Feng, Siyuan Kong, Hongsheng Tan, Hongxi Xu, Cheng Huang

**Affiliations:** School of Pharmacy, Shanghai University of Traditional Chinese Medicine, 1200 Cailun Road, Shanghai 201203, China

## Abstract

The aim of this study was to assay the effects of* Coreopsis tinctoria* Nutt. flower extracts on hyperglycemia of diet-induced obese mice and the underlying mechanisms.* Coreopsis tinctoria* flower was extracted with ethanol and water, respectively. The total phenol, flavonoid levels, and the constituents of the extracts were measured. For the animal experiments, C57BL/6 mice were fed with a chow diet, high-fat diet, or high-fat diet mixed with 0.4% (w/w) water and ethanol extracts of* Coreopsis tinctoria* flower for 8 weeks. The inhibitory effects of the extracts on *α*-glucosidase activity and the antioxidant properties were assayed* in vitro*. We found that the extracts blocked the increase of fasting blood glucose, serum triglyceride (TG), insulin, leptin, and liver lipid levels and prevented the development of glucose tolerance impairment and insulin resistance in the C57BL/6 mice induced by a high-fat diet. The extracts inhibited *α*-glycosidase activity and increased oxidant activity* in vitro*. In conclusion,* Coreopsis tinctoria* flower extracts may ameliorate high-fat diet-induced hyperglycemia and insulin resistance. The underling mechanism may be via the inhibition of *α*-glucosidase activity. Our data indicate that* Coreopsis tinctoria* flower could be used as a beverage supplement and a potential source of drugs for treatment of diabetics.

## 1. Introduction

Diabetes is a metabolic disease characterized by hyperglycemia, reduced glucose tolerance, and insulin-releasing abnormalities. The latest estimates released by the International Diabetes Federation indicate that a staggering 382 million people suffered from diabetes in 2014. The inhibitor of *α*-glucosidase, a carbohydrate-hydrolyzing enzyme, has been identified as an effective therapy for type II diabetes [1]. The production and absorption of glucose can be restricted or delayed through the inhibition of *α*-glucosidase. Compared with other oral medications of diabetes and insulin therapy, *α*-glucosidase inhibitors have the most notable characteristics of being able to effectively treat or prevent high blood glucose from diabetic or prediabetic patients [2]. In addition, *α*-glucosidase inhibitors have other effects, such as regulation of lipogenesis [3] and inhibiting viral infection [4]. Therefore, *α*-glucosidase inhibition is a safe and efficient antidiabetic therapy, and it has attracted a lot of attention in recent years [5].

Oxidative stress is closely related to diabetes, and it plays an important role in the development of diabetes and is involved in the whole process of diabetes and its complications [6, 7]. An antidiabetic drug with antioxidant properties may improve high blood glucose and related disorders.

*Coreopsis tinctoria* Nutt. (Golden tickseed) is a plant native to North America that is cultured worldwide. The flower of* Coreopsis tinctoria* is used as a beverage and as a nutraceutical for reducing bodyweight, high serum lipids, and blood sugar, as well as for treating cardiovascular disease, hypertension, diarrhea, and emesis in Native American and traditional Chinese and Portuguese medicine [8–10]. Recent studies have found that the extracts and flavonoids from* Coreopsis tinctoria Nutt*. flower (CTF) displayed vasorelaxant properties via inhibition of calcium movement through cell membranes in rat thoracic aortic rings [11]. The flavonoid-rich fraction of the* Coreopsis tinctoria* flower increases glucose tolerance through pancreatic function recovery in streptozotocin-induced diabetic rats [2, 8], improves high-fat diet-induced hepatic insulin resistance in rats through the inhibiting of gluconeogenic pathway key proteins glucose-6-phosphatase and phosphoenolpyruvate carboxykinase [12], and protects mouse insulinoma MIN6 cells from tert-butyl-hydroperoxide oxidative stress and cytokine-induced injury [9]. Coreosides A, B, C, and D, C14-polyacetylene glycosides of* Coreopsis tinctoria*, display anti-inflammatory activity against cyclooxygenase-2 [13]. Although CTF has been well studied for its various health benefits, there is little experimental data about its effects and underlying mechanisms on hyperglycemia and antioxidant properties.

In the present study, we identified that the extracts of CTF could lower blood glucose and serum triglyceride levels in high-fat diet-induced obese mice and displayed *α*-glycosidase inhibitory and antioxidant activity* in vitro*.

## 2. Materials and Methods

### 2.1. Chemicals and Diet

*Coreopsis tinctoria* flowers were obtained from Xinjiang, China. The* Coreopsis tinctoria* flowers (EEC) were weighed and were extracted in a reflux extraction system using 10 times of 95% ethanol or water (w/v) for 2 hours twice. The extracts were concentrated with a rotary evaporator under reduced pressure at 50°C and freeze-dried. High-fat diet (60% of calories derived from fat, D12492) and chow diet (10% of calories derived from fat, D12450B) were purchased from Research Diets (New Brunswick, NJ, USA).

### 2.2. Animals and Treatment

The animal protocols used in this study were approved by the Shanghai University of Traditional Chinese Medicine. Female C57BL/6 mice were purchased from the SLAC Laboratory (Shanghai, China). All animals were kept under controlled temperature (20–25°C) and on a 12-h light/12-h dark cycle. For induction of metabolic disorders, C57BL/6 mice with corresponding age and body weight were randomly separated into different groups and then placed on a high-fat (HF) diet (60% of calories derived from fat, 5.24 Kcal/gm, Research Diets, D12492) or the HF diet plus 0.4% (the highest dose that does not alter food consumption) CT extracts, while the low-calorie diet was the equivalent of a chow diet control (10% of calories derived from fat, 3.85 Kcal/gm, Research Diets, D12450B). The diet treatment was started at 4 weeks of age and lasted for 8 weeks. Twenty-four-hour food intake was recorded in both treated and control groups during the treatment. The experimental results did not lead to any change in the daily food intake compared to the control.

### 2.3. Rectal Temperature Measurement

The rectal temperature of mice was examined with a rectal probe attached to a digital thermometer (Physitemp, NJ, USA).

### 2.4. Intraperitoneal Glucose Tolerance Test (GTT) and Insulin Tolerance Tests (ITT)

At the end of the treatment, C57BL/6 mice were fasted overnight (12 h). The baseline glucose values (0 min) before the injection of glucose (1 g/kg body weight) were determined by means of collecting blood samples from the tail vein. In addition, blood samples were collected at regular intervals (15, 30, 60, 90, and 120 min) following the injection of glucose.

For insulin tolerance test, the baseline glucose values (0 min) with no fasting before the injection of insulin (0.75 U/kg body weight) (Sigma, St. Louis, MO, USA) were determined. Further blood samples were tested at regular intervals (15, 30, 60, 90, and 120 min) following the injection of insulin.

### 2.5. Enzyme-Linked Immunosorbent Assay (ELISA)

Serum insulin, leptin, and adiponectin levels were measured with ELISAs for mouse insulin (ALPCO Diagnostics, Salem, NH, USA), adiponectin ELISA Kit (R&D Systems, Oxon, UK), and leptin (AdipoGen, Seoul, Korea) according to the manufacturer's instructions.

### 2.6. Serum Chemistry Analysis

At the end of the treatment, the mice were fasted overnight, and the blood samples were collected by cardiac puncture with anesthesia (20% urethane, w/v). Then the serum samples were separated through centrifuging at 3000 rpm for 15 min. The serum triglyceride (TG), total cholesterol (TC), HDL cholesterol (HDL-c), and LDL cholesterol (LDL-c) were examined using a Hitachi 7020 Automatic Analyzer (Hitachi, Tokyo, Japan) with 100 *μ*L of heart blood serum.

### 2.7. Liquid Chromatograph-Mass Spectrometer (LC-MS) Analysis

The chromatographic analyses were carried out using Ultra Performance Liquid Chromatography & Triple Quadrupole Mass Spectrometer (UPLC-3QMS) (ACQUITY UPLC & SCIEX SelexION Triple Quad*™* 5500 System). The following gradient was applied (flow rate: 0.4 mL/min; 0–0.5 min: 95.0% A + 5.0% B; 0.5–7 min: 95.0% A + 5.0% B; 7–13 min: 80.0% A + 20.0% B; 13–13.5 min: 70.0% A + 30.0% B; 13.5–14.5 min: 50.0% A + 50.0% B; 14.5–15 min: 15.0% A + 85.0% B; 15–18 min onward: 100% B; 18–20 min: 95.0% A + 5.0% B. The injection volume was 5.0 *μ*L). The MS conditions were polarity ES^−^; capillary: 2800 V; cone voltage: 55.0 V; source temperature: 110°C; desolvation temperature: 350°C; cone gas flow: 50.0 L/Hr; desolvation gas flow: 600.0 L/Hr; scan time: 0.300 s; interscan time: 0.020 s; collision energy: 4.0 eV. The HPLC eluate full-scan MS mass spectra were acquired in the mass range 80–1500* m/z* in electrospray mode with negative ions [M-H].

### 2.8. *α*-Glycosidase Inhibitory Activity

The *α*-glycosidase inhibitory activity of the extracts was determined according to the method described by Apostolidis and Lee with slight modifications [14]. A mixture of 630 *μ*L of sample, including 40 *μ*L of samples or acarbose, 20 *μ*L of 3 mM glutathione diluent with ddH_2_O, 50 *μ*L of 5 mM p-nitrophenyl-*β*-galactoside (PNPG) solution, 500 *μ*L of 0.1 M phosphate buffer (pH 6.9), and 20 *μ*L of *α*-glycosidase solution (1 U/mL) was added to each centrifuge tube and was incubated at 37°C for 20 min. Next, 40 *μ*L of reaction mixtures was placed into 96-well plates, and 160 *μ*L of 0.1 M sodium carbonate solution was added to terminate the reaction. Absorbance was recorded at 400 nm using microplate reader. Acarbose was used as the positive control. The *α*-glycosidase inhibitory activity is expressed as the inhibition percentage and was calculated as follows: inhibition (%) = (1 − Δ*A*_sam_/Δ*A*_con_) × 100%. Δ was defined as absorbance of the sample or the control.

### 2.9. Antioxidant Capacity Assay

The free radical-scavenging activities of EEC and WEC were assayed by using kits from Biyuntian (Shanghai, China) for 2,2-diphenyl-1-picrylhydrazyl (DPPH) radical-scavenging activity, 2,2′-azino-bis 3-ethylbenzothiazoline-6-sulphonic acid (ABTS) radical-scavenging activity, and Ferric-reducing ability power (FRAP) according to our previous report [15].

### 2.10. Statistical Analysis

The data are expressed as mean ± SD. Data analyses of the control and the prevented groups were performed using the SPSS 21.0 for Windows statistical program. Differences were regarded as statistically significant when *P* < 0.05.

## 3. Results

### 3.1. Flavonoid Content Analysis and LC-MS Analysis of EEC and WEC

**Figure 1:**
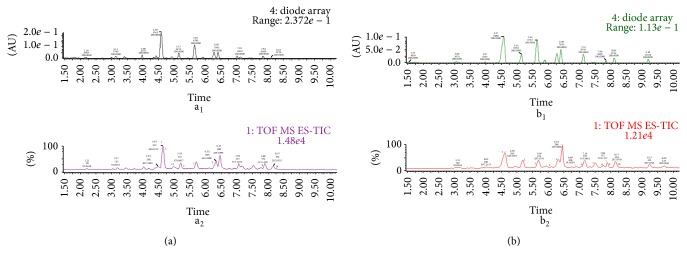
LC-MS chromatographic profile of CTF extracts. (a) Ms spectra at 280 nm (a_1_) and absorbance spectra (a_2_) of the WEC. (b) Ms spectra at 280 nm (b_1_) and absorbance spectra (b_2_) of the EEC. 1: 3′,5,5′,7-Tetrahydroxyflavanone-O-hexoside; 2: Quercetagitin-7-O-glucoside; 3: Okanin; 4: 3,4′,5,6,7-Pentahydroxyflavanone; 5: Luteolin-7-O-glucoside; 6: Chlorogenic Acid; 7: Luteolin; 8: Coreopsin; 9: Dicaffeoylquinic acid; 10: 3′,5,5′,7-Tetrahydroxyflavanone; 11: Coreopsin.

We characterized the constituents of the extracts using LC-MS.  Figure 1 shows the spectra of EEC and WEC. The peaks were interpreted by considering the parent and daughter ions associated with each peak and by consulting published results [16, 17]. The interpretations are summarized in Table 1 and showed that both EEC and WEC were rich in flavonoid compounds, and EEC contained more constituents. These data are in agreement with the previous reports [13, 18].

### 3.2. EEC and WEC Block the Increase of Fasting Blood Glucose and Prevent Insulin Resistance in Mice Induced by a High-Fat Diet

**Figure 2:**
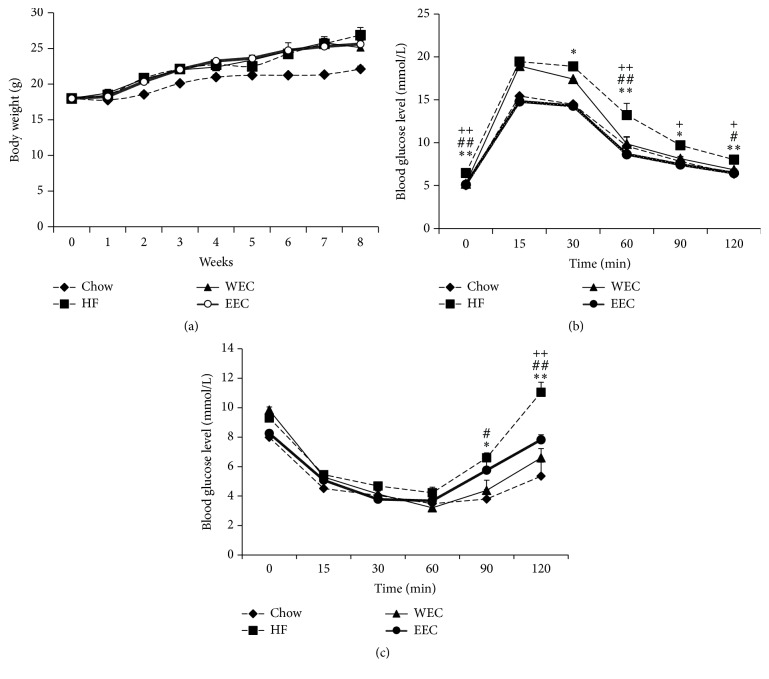
EEC and WEC prevent metabolic disorders included in C57BL/6 mice by a high-fat diet. (a) Body weight. (b) Intraperitoneal glucose tolerance test. The mice were fasted for 12 hours, and the fasting blood glucose was measured, and then 1 g glucose/1 kg body weight was given by intraperitoneal injection. And the blood glucose levels at 15, 30, 60, 90, and 120 min were tested. (c) Intraperitoneal insulin tolerance test. The blood glucose level was tested at 0, 15, 30, 60, 90, and 120 min following the intraperitoneal injection of insulin (0.75 U/kg body weight). The data are expressed as mean ± SEM. *N* = 7 for all groups, Repeated measurement, SPSS 21.0. ^*∗*^ *P* < 0.05, ^*∗∗*^ *P* < 0.01: the Chow group versus the HF group; ^#^ *P* < 0.05, ^##^ *P* < 0.01: the WEC group versus the HF group; ^+^ *P* < 0.05, ^++^ *P* < 0.01: the EEC group versus the HF group.

Female C57BL/6 mice were fed with a chow diet (Chow) or a high-fat (HF) diet or a HF diet mixed with 0.4% EEC and WEC, respectively, for 8 weeks. At the end of the treatment, the body weight of the HF control was significantly higher than that of the chow diet-fed mice. Although the food intake in the EEC and WEC groups was lower than that in the HF group (Table 2), the body weight of the EEC and WEC groups was similar to that of the HF group (Figure 2(a)).

Next, the fasting blood glucose levels in the mice were tested. The HF group showed significantly higher fasting blood glucose concentrations compared to the Chow group (*P* < 0.05), while the fasting blood glucose levels were lower in EEC- and WEC-treated groups compared to that in the HF group (Table 2). We then analyzed the glucose tolerance in the mice. The blood glucose levels of the HF group at 15, 30, 60, 90, and 120 min following glucose injection were markedly increased when compared with those in the Chow group. EEC and WEC supplementation significantly blocked the increase of blood glucose levels at 60 min (Figure 2(b)), suggesting that EEC and WEC can prevent fasting blood glucose increase and glucose tolerance impairment in DIO mice.

Next, we performed an insulin tolerance test. As shown in Figure 2(c), the blood glucose levels of the HF group were markedly increased at 0, 15, and 90 min following insulin injection compared to those of the Chow group. The blood glucose levels at 0, 90, and 120 min in EEC-treated mice were lower than that in the HF group. Similarly, the blood glucose levels at 90 and 120 min in WEC group were also lower, indicating that EEC and WEC may improve glucose tolerance in DIO mice.

We then assayed the serum lipid profiles of the mice. Although the serum levels of TC, LDL, and HDL were not changed significantly in the WEC and EEC mice (Table 2), the TG contents were lower in the EEC-treated mice, but not the WEC-treated mice (Table 2), indicating that EEC may prevent the increase of triglyceride in DIO mice.

### 3.3. Effects of EEC and WEC on Serum Insulin, Adiponectin, and Leptin Levels in DIO Mice

**Figure 3:**
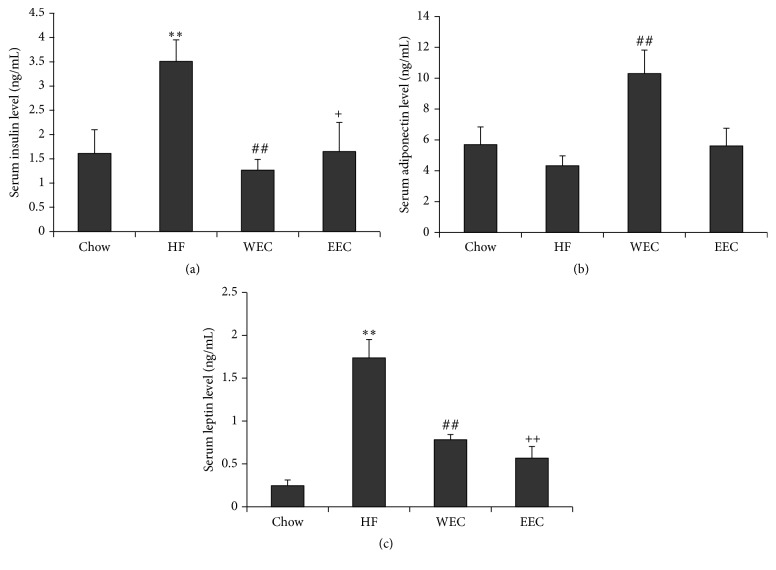
Effects of CTF extracts on serum insulin, adiponectin, and leptin levels. (a) Insulin; (b) adiponectin; (c) leptin. Levels in high-fat diet. The data are expressed as mean ± SEM. *N* = 7 for all groups, One-way ANOVA, SPSS 21.0. ^*∗∗*^ *P* < 0.01: the Chow group versus the HF group; ^##^ *P* < 0.01: the WEC group versus the HF group; ^+^ *P* < 0.05, ^++^ *P* < 0.01: the EEC group versus the HF group.

Since DIO mice may develop hyperinsulinemia, we assayed the serum insulin levels of mice. The serum insulin levels of the HF group were significantly higher than those of the Chow mice (Figure 3(a)). Both EEC and WEC treatments markedly prevented the increase of serum insulin levels when compared with the HF group (Figure 3(a)). These results suggest that the EEC and WEC may prevent the development of insulin resistance in mice fed the HF diet. As adiponectin and leptin can reverse insulin resistance, the serum levels of adiponectin and leptin were measured. Although the adiponectin concentrations of the HF group were not significantly reduced when compared to the Chow group, the WEC group had significantly higher levels (Figure 3(b)); however, EEC did not change the serum adiponectin levels. The serum leptin levels of the HF group were higher compared with that of the Chow group. EEC and WEC treatments significantly blocked the enhancement of leptin levels compared to DIO mice (Figure 3(c)). These results suggest that EEC and WEC may improve hyperinsulinemia and hyperleptinemia and protect from the decrease of serum adiponectin levels in DIO mice.

### 3.4. EEC and WEC Inhibit *α*-Glycosidase Activity* In Vitro*

**Figure 4:**
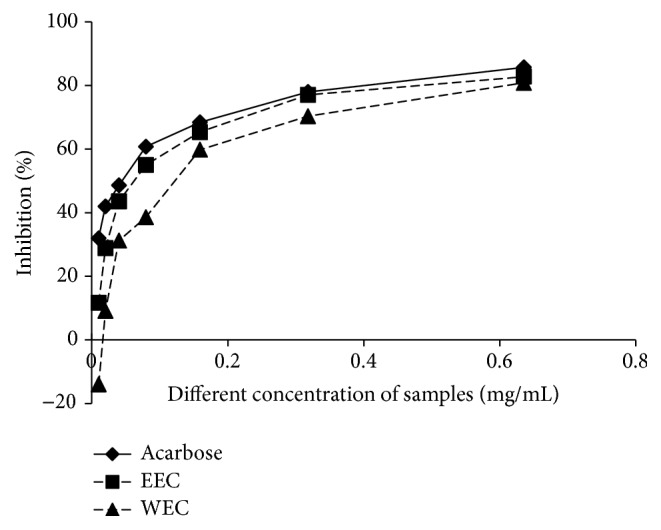
Inhibitory activities of CTF extracts against *α*-glycosidase. The experiment was performed with triplicates. Two independent experiments showed similar results.

To explore the underlying mechanism of blood glucose-lowering effects of the extracts, the inhibitory effects of EEC and WEC on *α*-glycosidase activity were assayed* in vitro*. EEC and WEC induced a concentration-dependent suppression on *α*-glycosidase activity: 11.59% to 82.72% for EEC at the concentration of 0.01 mg/mL to 0.63 mg/mL and 0% to 80.82% for WEC at 0.01 mg/mL to 0.63 mg/mL, compared to the inhibitory rate of 85.67% for the *α*-glycosidase inhibitor, acarbose (Figure 4). The IC_50_ of acarbose, EEC, and WEC were at the concentrations of 0.038 ± 0.003, 0.067 ± 0.004, and 0.125 ± 0.005 mg/mL, respectively (Figure 4).

### 3.5. Antioxidant Capacities of EEC and WEC

**Figure 5:**
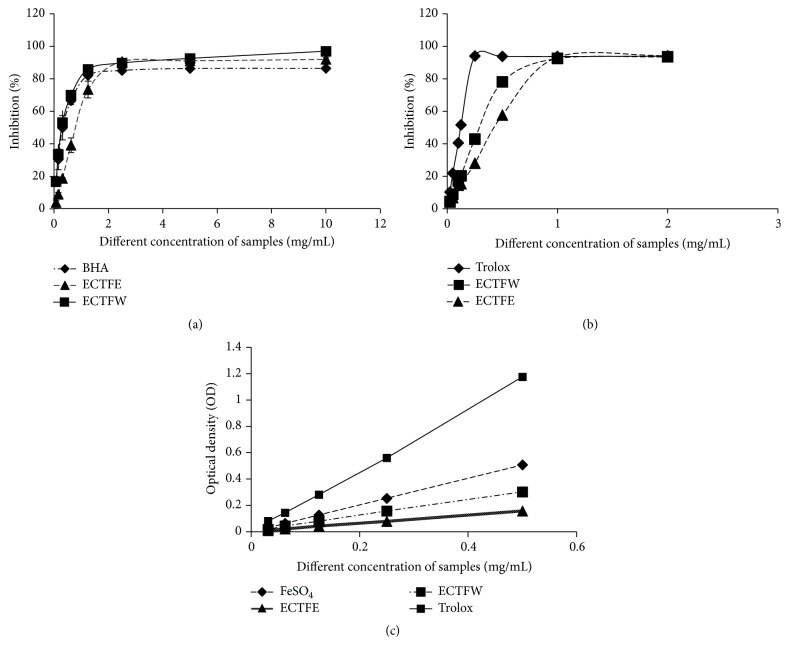
Antioxidant ability of CTF extracts. (a) DPPH free radical-scavenging activity. (b) ABTS radical-scavenging capacity. (c) Ferric-reducing ability power (FRAP) assay. The experiment was performed with triplicates.

The antioxidant activities of EEC and WEC were tested using the DPPH free radical-scavenging activity assay, ABTS radical-scavenging capacity assay, and the Ferric-reducing ability power (FRAP) assay. The radical-scavenging activities of WEC and EEC had DPPH IC_50_ values of 0.298 ± 0.051 mg/mL and 0.759 ± 0.067 mg/mL, respectively (Figure 5(a)). The activities of EEC and WEC were analyzed using the ABTS and FRAP assays (Figures 5(b) and 5(c)). As shown in (Figure 5(b)), the total antioxidant activities of EEC and WEC were equivalent to 0.268 ± 0.003 mg/mL and 0.383 ± 0.046 mg/mL of Trolox in the ABTS assay, suggesting a strong antioxidant activity of CTF.

## 4. Discussion

The dried* Coreopsis tinctoria* flower is used in beverages because of its flavor and its potential health benefits. In the present study, we verified that the extracts of* Coreopsis tinctoria* flower could lower blood glucose levels through the inhibition of *α*-glycosidase activity and via its antioxidant properties.

Previous studies have shown that CTF is rich in flavonoids and polyphenols [13, 18]. Similarly, we found that EEC and WEC contain 27.28% and 36.2% total flavonoids, respectively. LC/MS revealed that the highly occurring constituents in the extracts were the flavonoids and polyphenols.

*Coreopsis tinctoria* flower has traditionally been used to control diabetes in Portugal [8, 9]. It has been reported that* Coreopsis tinctoria* flower infusion reduced blood glucose levels in glucose-intolerant Wistar rats [2] and improved high-fat diet-induced hepatic insulin resistance in rats through the inhibiting of gluconeogenic pathway [12] and glucose tolerance through pancreatic function recovery in streptozotocin-induced diabetic rats [8]. This function of* Coreopsis tinctoria* flower is probably through promoting the impaired pancreatic cells.

In the present study, we identified that the extracts of* Coreopsis tinctoria* flower may prevent the increase of blood glucose levels and the development of insulin resistance in mice induced by a HF diet. This effect was further supported by the data of serum insulin, adiponectin, and leptin levels in the DIO mice, which were reversed by the treatment of CTF extracts. This diabetic model is caused by insufficient insulin sensitivity [19], but not pancreas damage. Thus, the pancreatic protective function cannot explain how the extracts lower blood glucose level in the insulin-resistant animals. Taken together, our data suggest that CTF extracts could improve insulin resistance in DIO mice.

*α*-Glucosidase is an intestinal enzyme that digests carbohydrates to release glucose [20]. Inhibition of *α*-glucosidase may suppress the rate of digestion of carbohydrates and block the absorption of glucose from food [21, 22]. In diabetic patients, *α*-glucosidase inhibitors may decrease blood glycated hemoglobin A1C level [23, 24]. Inhibitors of *α*-glycosidase, such as acarbose [25–27], have been identified as effective antidiabetic drugs for type 2 diabetes and prediabetes. Both EEC and WEC displayed significant inhibitory effects on *α*-glucosidase activities* in vitro* that were similar to acarbose (Figure 5), suggesting that the extracts contain an *α*-glucosidase inhibitor. We assumed that the active compound is flavonoid, because only about 1% of flavonoid can be absorbed in intestine. However, which constituent is involved in the *α*-glucosidase inhibitory effect remains uncertain because it is difficult to obtain the compounds.

We also observed that the EEC prevented the increase of serum triglyceride contents in DIO mice. It has been reported that the administration of acarbose also improves postprandial hyperlipidemia [25, 28]. Long-term treatment with acarbose decreases serum triglycerides in diabetic patients [29, 30], suggesting that inhibition of *α*-glucosidase may improve lipid disorders. However, we cannot exclude that other mechanisms may be involved in the action.

As abundant evidence has implied, oxidative stress is associated with a wide range of diseases [6, 7]. Antioxidant therapy is assumed to be a possible intervention to treat diabetes and its complications [31]. Antidiabetic drugs with antioxidant capacity could be beneficial to diabetic patients. Recent studies have shown that the flavonoids from* Coreopsis tinctoria* flower displayed antioxidant activities [18, 32]. In the present study, the antioxidant activities of EEC and WEC were evaluated through three different methods. All methods showed that both extracts have strong antioxidant activity.

In conclusion, the extracts of the* Coreopsis tinctoria* flower were shown to prevent the increase of blood glucose levels and glucose tolerance impairment of mice induced by a high-fat diet. The hypoglycemic effects of the extracts may be exerted through the inhibition of *α*-glucosidase activity and the antioxidant properties. Our study provides evidence for the use of* Coreopsis tinctoria* flower to treat or prevent diabetes through the multiple mechanisms and suggests that the* Coreopsis tinctoria* flower may be used as a nutriceuticals or potential source of drug for diabetes.

## Figures and Tables

**Table 1:** Chemical composition in EEC and WEC identified by LC-MS.

Peak	*R*_*t*_ (min)	Constituents	[M-H]^−^	Daughter ions	EEC	WEC
1	4.60	3′,5,5′,7-Tetrahydroxyflavanone-O-hexoside	449.1057	287 [M-H-hexose]^−^	√	
2	4.97	Coreopsin	433.1132	271 [M-H-glucose]^−^		√
3	5.17	Quercetagitin-7-O-glucoside	479.0811	317 [M-H-glucose]^−^	√	√
4	5.69	Okanin	287.0546	151 (^1,3^A^−^), 135 [^1,3^A^−^-16]^−^	√	√
5	5.93	3,4′,5,6,7-Pentahydroxyflavanone	303.0501	285 [M-H-H_2_O]^−^	√	
6	6.33	Luteolin-7-O-glucoside	447.0919	285 [M-H-glucose]^−^	√	√
7	7.16	Chlorogenic Acid	353.0861	191 [M-H-caffeic acid]^−^	√	
8	7.50	Luteolin	285.0398	133 [^1,3^A^−^-18]^−^	√	
9	7.76	Coreopsin	433.1118	271 [M-H-glucose]^−^, 135 [^1,3^A^−^-16]^−^	√	
10	7.88	Dicaffeoylquinic Acid	515.1171	353 [M-H-caffeic acid]^−^	√	√
11	9.23	3′,5,5′,7-Tetrahydroxyflavanone	287.0544	151 (^1,3^A^−^)	√	√

*R*_*t*_: retention time (minute), [M-H]^−^: the primary Mass Spectrometry.

**Table 2:** The food intake, body temperature, and serum glucose and lipid profiles in the mice.

	Chow (Ave ± SD)	HF (Ave ± SD)	WEC (Ave ± SD)	EEC (Ave ± SD)
Food intake (g/d)	2.7 ± 0.31^*∗∗*^	3.1 ± 0.34	2.3 ± 0.15^##^	2.4 ± 0.22^++^
Rectal temperature (°C)	37.7 ± 0.466	37.2 ± 0.311	37.1 ± 0.285	37.1 ± 0.462
Fasting glucose level (mmol/L)	5.05 ± 0.203^*∗∗*^	6.47 ± 0.283	5.24 ± 0.269^##^	5.13 ± 0.190^++^
Serum TC (mmol/L)	2.51 ± 0.248^*∗∗*^	3.25 ± 0.436	3.03 ± 0.248	2.88 ± 0.172
Serum TG (mmol/L)	0.756 ± 0.138^*∗∗*^	1.11 ± 0.131	1.01 ± 0.137	0.920 ± 0.194^+^
Serum LDL (mmol/L)	0.803 ± 0.0446^*∗∗*^	0.897 ± 0.116	0.853 ± 0.0814	0.832 ± 0.0895
Serum HDL (mmol/L)	1.21 ± 0.106^*∗∗*^	1.47 ± 0.117	1.66 ± 0.130	1.62 ± 0.151

The data are expressed as mean ± SD. *N* = 7 for each group. ^*∗∗*^ *P* < 0.01: the Chow group versus the HF group; ^##^ *P* < 0.01: the WEC group versus the HF group; ^+^ *P* < 0.05: the EEC group versus the HF group and ^++^ *P* < 0.01: the EEC group versus the HF group.

## Additional Points

Chemical compounds are the following: Coreopsin (PubChem CID: 25245366); Okanin (PubChem CID: 5281294); Dicaffeoylquinic Acid (PubChem CID: 6474640); Luteolin-7-O-glucoside (PubChem CID: 5280637); Chlorogenic Acid (PubChem CID: 1794427); Luteolin (PubChem CID: 5280637); Coreopsin (PubChem CID: 25245366); 3′,5,5′,7-Tetrahydroxyflavanone (PubChem CID: 11483087); and Pentahydroxyflavanone (PubChem CID: 440836).
